# A novel hypomorphic splice variant in EIF2B5 gene is associated with mild ovarioleukodystrophy

**DOI:** 10.1002/acn3.51131

**Published:** 2020-08-15

**Authors:** Agustí Rodríguez‐Palmero, Agatha Schlüter, Edgard Verdura, Montserrat Ruiz, Juan José Martínez, Isabelle Gourlaouen, Chandran Ka, Ricardo Lobato, Carlos Casasnovas, Gérald Le Gac, Stéphane Fourcade, Aurora Pujol

**Affiliations:** ^1^ Neurometabolic Diseases Laboratory Bellvitge Biomedical Research Institute (IDIBELL) L'Hospitalet de Llobregat 08908 Spain; ^2^ Pediatrics Department University Hospital Germans Trias i Pujol Badalona 08916 Spain; ^3^ Center for Biomedical Research on Rare Diseases (CIBERER) ISCIII Madrid Spain; ^4^ INSERM U1078 Brest France; ^5^ Laboratory of Excellence GR‐Ex Paris France; ^6^ Laboratoire de Génétique Moleculaire et Histocompatibilité CHRU de Brest Hôpital Morvan Brest France; ^7^ Neurology Department Hospital Universitario Infanta Sofía San Sebastián de los Reyes 28703 Spain; ^8^ Neuromuscular Unit Neurology Department Hospital Universitari de Bellvitge L'Hospitalet de Llobregat 08908 Spain; ^9^ Université Bretagne Loire Université de Bretagne Occidentale IBSAM Brest France; ^10^ Catalan Institution of Research and Advanced Studies (ICREA) Barcelona Spain

## Abstract

**Objective:**

To identify the genetic cause in an adult ovarioleukodystrophy patient resistant to diagnosis.

**Methods:**

We applied whole‐exome sequencing (WES) to a vanishing white matter disease patient associated with premature ovarian failure at 26 years of age. We functionally tested an intronic variant by RT‐PCR on patient’s peripheral blood mononuclear cells (PBMC) and by minigene splicing assay.

**Results:**

WES analysis identified two novel variants in the *EIF2B5* gene: c.725A > G (p.Tyr242Cys) and an intronic noncanonical mutation (c.1156 + 13G>A). This intronic mutation resulted into generation of various isoforms both in patient’s PBMC and in the minigene splicing assay, showing that ~20% residual wild‐type isoform is still expressed by the intronic‐mutated allele alone, concordant with an hypomorphic effect of this variant.

**Conclusion:**

We report two novel variants in *EIF2B5*, one of them a noncanonical intronic splice variant, located at a +13 intronic position. This position is mutated only in 0.05% of ClinVar intronic mutations described so far. Furthermore, we illustrate how minigene splicing assay may be advantageous when validating splice‐altering variants, in this case highlighting the coexistence of wild‐type and mutated forms, probably explaining this patient’s milder, late‐onset phenotype.

## Introduction

Vanishing white matter disease (VWMD; OMIM #603896) is a leukodystrophy caused by recessive mutations in any of the five genes encoding subunits of translation initiation factor EIF2B. Manifestations usually start between late infancy and early childhood, and mainly consist of pyramidal and cerebellar signs with mental decline and episodes of acute deterioration following stressors. However, 15% of cases are adult‐onset forms associated with a more benign course and fewer decompensations.^1^ Infrequently, VWMD can appear associated with premature ovarian failure (POF), a clinical syndrome that has been called ovarioleukodystrophy.^2^ Although *EIF2B1‐5* mutations are the main cause of leukodystrophy with POF, other genes have been described.^3^

More than 120 mutations have been reported in >250 patients with an EIF2B‐related disorder, mostly in *EIF2B5* and *EIF2B2*.^4^ No mutational hotspots have been found, although some recurrent mutations seem to occur in paired cytosine/guanine (CpG) dinucleotides. The vast majority of pathogenic mutations are missense, whereas truncating mutations (frameshifts, nonsense, splice site mutations) are rare and have been reported only in compound‐heterozygous state,^5^ indicating that total loss of function may be incompatible with life.

Here, we report a patient with an adult‐onset ovarioleukodystrophy carrying two novel disease‐causing variants in *EIF2B5* identified by WES, one of which is an intronic mutation leading to activation of a cryptic splice donor site. The impact of this rare, noncanonical splice variant on *EIF2B5* pre‐mRNA processing was evaluated both in cDNA from patient’s PBMC, and using minigene splicing reporter assays. These experiments revealed residual wild‐type splicing, probably accounting for the mild late‐onset clinical phenotype in this patient.

## Methods

### Participant and ethics

Blood was processed by centrifugation within 2 h of collection using a gradient of Histopaque to separate plasma, erythrocytes, and PBMC. Plasma and PBMC were stored at −80°C. The use of all samples was approved by the Clinical Research Ethics Committee of the Bellvitge University Hospital (PR076/14). Informed written consent was obtained from all patients and control individuals.

### Exome sequencing and variant calling

In‐solution exome capture was performed using the SeqCap EZ Human Exome Kit v3.0 (Roche Nimblegen, USA) with 100‐bp paired‐end read sequences generated on a HiSeq2000 (Illumina, Inc. USA) in the Centro Nacional de Análisis Genómico in Barcelona (CNAG). Sequence processing was carried out with BWA aligner, the Genome Analysis Toolkit (GATK), SAMtools, and Picard Tools as previously described.^6^

More information regarding methods are detailed in the Data S1.

## Results

### Clinical findings

**Figure 1:**
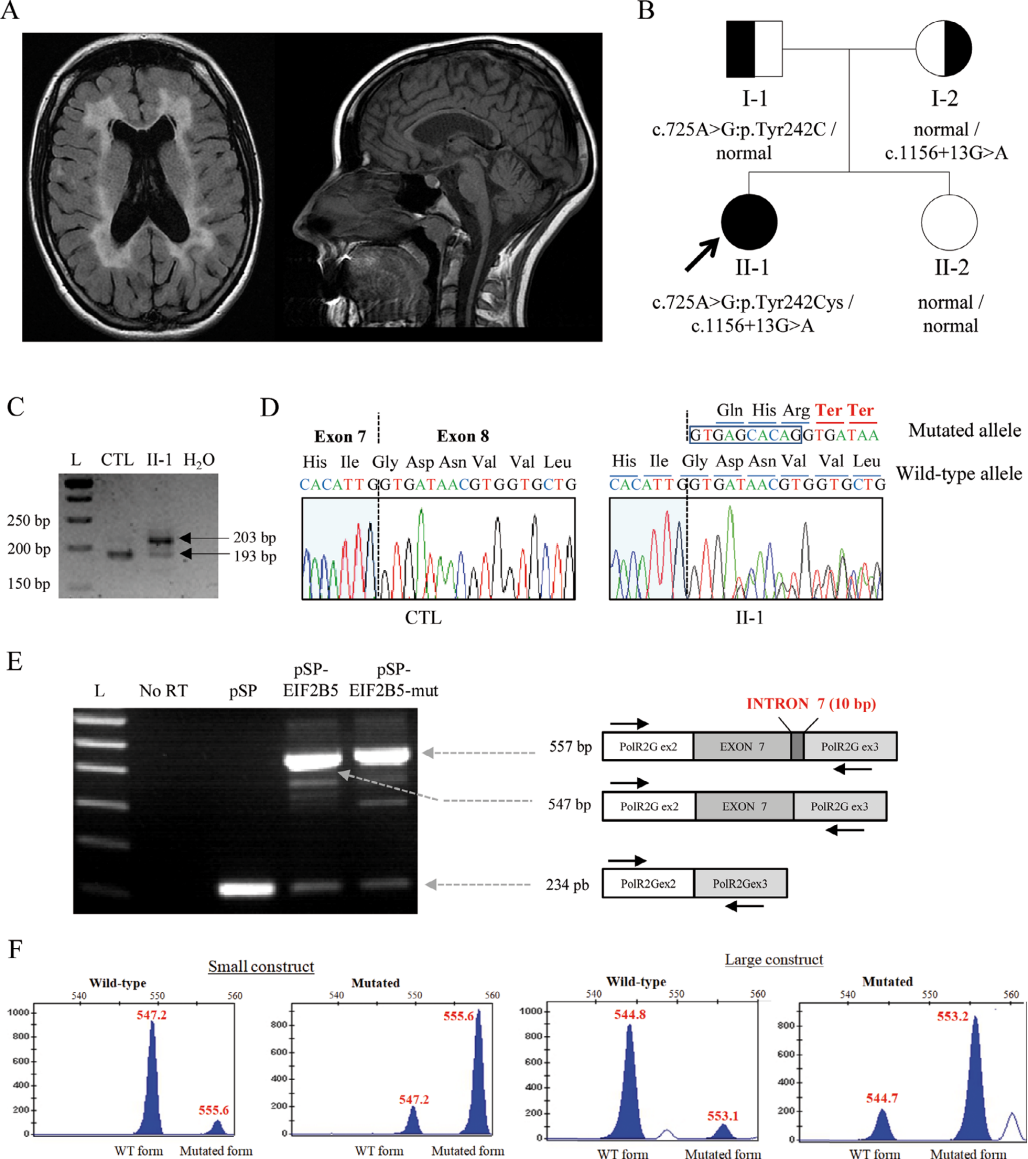
Clinical and genetic features. (A) Periventricular T2 white matter hyperintensities with small areas of cystic degeneration, and corpus callosum and mild cerebellar atrophy. (B) Family tree and cosegregation analysis of *EIF2B5* variants. (C) PCR amplified products of cDNA from II‐1 and a healthy individual (CTL) resolved on a 4% agarose gel. L: Ladder (GeneRuler 50bp DNA ladder). (D) Partial sequence chromatograms of cDNA from CTL and II‐1. Nucleotide and amino acid sequences of normal and mutated alleles are written above of each chromatogram. (E) RT‐PCR from transfected HEK 293T17 and sequencing profiles of the products with retention of intron 7. Arrows indicate the relative positions of forward and reverse primers; the different constructs and sizes obtained are mentioned in the figure. L: Ladder; No RT: No Reverse Transcriptase; pSP: pSplicePOLR2G vector alone, pSP‐EIF2B5: WT *EIF2B5* cloned into pSP and pSP‐EIF2B5‐mut: intronic variant of *EIF2B5* cloned into pSP. (F) Capillary electrophoresis of fluorescent RT‐PCR products from wild‐type and derived mutant minigenes, for the short and large constructs.

A 26‐year‐old woman was referred to the neurology department because of a leukoencephalopathy detected during the study of amenorrhea with hyperprolactinemia (108 ng/mL). She was born from nonconsanguineous parents and she had one asymptomatic sister. Physical exam showed right hand clumsiness, dystonic left foot postures, and generalized hyperreflexia in absence of cognitive, behavior or psychiatric symptoms. MRI evidenced T2 periventricular and pontine white matter hyperintensities with malacic areas in the frontal and atrial horns. Cortical‐subcortical, spinal, and corpus callosum atrophy were also reported (Fig. 1A). Magnetic resonance spectroscopy was unremarkable. In the next 5 years, she developed unsteady gait, more evident pyramidal signs and lower limb paresthesia and cramps. Her cognitive level remained normal and she did not develop seizures. MRI performed four years after the initial one did not show significant changes. Routine hematology and clinical chemistry tests, as well as thyroid and adrenal function, were normal. Nerve conduction tests and electromyography were normal, whereas visual‐evoked potentials showed bilateral latency enlargement of the P100 wave and somatosensory‐evoked potentials exhibited bilaterally prolonged latencies with normal amplitudes.

### WES analysis

Whole‐exome sequencing (WES) revealed two suspicious novel variants in *EIF2B5*. The first one is a missense variant (NM_003907:c.725A > G; p.Tyr242Cys) referenced in dbSNP database as rs750767613, and has a frequency of 3,98.10e‐6 in gnomAD database, with 0 homozygotes. This ultrarare missense mutation alters a highly conserved residue, and is predicted to be damaging by SIFT and PolyPhen‐2. The second variant is intronic (NM_003907:c.1156 + 13G>A) and is predicted by different algorithms to increase the use of a cryptic donor splice site located at positions + 11/+12 (NNSplice, MaxEntScan, FSplice). Conversely, Human Splicing Finder and SpliceView did not predict a significant impact on splicing (Table S1). This variant is absent from gnomAD database. Cosegregation analysis revealed that the patient inherited these variants in *trans* (Fig. 1B). Despite their rare frequency, cosegregation, conservation of affected amino acids, in silico predictions for missense and splicing variants, and the patient's highly concordant phenotype, these variants were classified as variants of unknown significance (VUS) according to the ACMG/AMP (American College of Medical Genetics and Genomics) guidelines for variant evaluation.^7^, ^8^

### In silico analysis of previously reported splicing variants

So far, 11998 single‐nucleotide variants probably affecting splicing and considered as pathogenic or likely pathogenic are listed in the ClinVar database. Splice‐site mutations are most commonly detected at the G(+1), T(+2), G(+5), A(−2), G(−1) canonical sequences of introns, as shown in Figure S1: +1 (4273 hits), −1 (2484), −2 (2181), +2 (1429), and +5 (551). Frequencies then decrease dramatically as distance from the acceptor/donor splice site increases. Only two variants have been reported in position +13: the A > G transition in intron 10 of the *MAPT* gene, associated with familial frontotemporal dementia with parkinsonism,^9^ and the C > T transition in *FGB* gene’s intron 6, associated with congenital afibrinogenemia.^10^

### Functional assays

Given c.1156 + 13G>A variant’s absence from databases and the discordant results of splicing predictors, we evaluated the functional significance of this variant in vitro. Reverse‐transcription PCR encompassing exons 7‐8 of the *EIF2B5* mRNA produced a single 193 bp product from the control samples, whereas two bands (193 and 203 bp) were observed in the patient’s sample. Sanger sequencing showed that the longest product included the first 10 nucleotides of intron 7. This 10‐bp insertion creates two adjacent premature termination codons (TGA and TAA), leading to a truncated protein (Fig. 1C and D). To confirm the association between the c.1156 + 13G>A *EIF2B5* variant and partial retention of intron 7, we performed a minigene splicing assay. This technique consists of the construction of an expression vector containing a minimal gene fragment encompassing the variant sequence of interest along with flanking intronic sequences, and then is transfected into cultured cells in order to evaluate splicing patterns.^11^, ^12^ This strategy allowed us to study the monoallelic effect of this *EIF2B5* variant compared to a wild‐type situation, and to evaluate more precisely the influence of the G > A change on the use of the identified donor cryptic splicing site. Two splicing reporter vectors were constructed in the background of the pSplice*POLR2G* plasmid^13^: a small construction containing *EIF2B5’s* exon 7 and flanking intronic sequences (143 bp of intron 6202 bp of intron 7), and a large construction encompassing *EIF2B5*’s exons 7 and 8 and the whole intron 7 sequence. Both vectors, with either the wild‐type or the mutated sequence, were transfected into HEK293T/17, U‐251MG, and COS‐7 cell lines. Results obtained from the smaller pSplice*POLR2G*‐*EIF2B5* construct in HEK293T cells show that the 10 intronic bp insertion was mainly, but not only, observed with the mutated c.1156 + 13G>A allele (Fig. 1E). Similar results were observed in U‐251MG and COS7 cells (Fig. S2A–C). Semi‐quantitative fluorescent RT‐PCR revealed a normal/abnormal‐splicing ratio of 0.9 for the wild‐type allele and 0.2 for the mutated allele (547 bp peak area/557 bp peak area) (Fig. 1F). Similar results were obtained from the larger pSplice*POLR2G*‐*EIF2B5* construct. These results indicate that: (1) a cryptic donor splicing site is active in *EIF2B5* intron 7 and is moderately used by the splicing machinery in different cell types; and (2) this cryptic splicing site is significantly more active in the c.1156 + 13G>A *EIF2B5* pre‐mRNA where it overcomes use of natural donor’s splicing site.

In conclusion, we have identified a new intronic variant in the *EIF2B5* gene which activates acryptic 5’ donor splice site of intron 7, probably leading to synthesis of a truncated protein (if not degraded by nonsense‐mediated mRNA decay). This splicing variant is hypomorphic, as it leads to a residual 20% of WT splicing, and should be considered pathogenic after applying the ACMG criteria.^7^, ^8^ In consequence, as VUS missense variant p.Tyr242Cys is *in trans* with the pathogenic intronic variant, this missense is reclassified as Likely Pathogenic^7^, ^8^ and our case is solved.

## Discussion

Splicing mutations represent approximately one‐third of disease‐causing mutations, most of them occurring in conserved consensus splice sites.^14^ However, the widespread use of high throughput technologies has increased the detection of variants in cryptic splice sites, which represent around 10% of total splicing mutations according to ClinVar data. In these cases, functional analysis is mandatory to confirm variant’s pathogenicity since, as in the case here reported, splicing predictors may provide discordant results. Our case also underscores the importance of thorough clinical evaluation guiding the bioinformatic analysis, to uncover variants in intronic regions. As incomplete coverage of intron‐exon boundaries in WES studies may hamper detection of intronic variants, WGS may still be required to unravel some cases.

To the best of our knowledge, only four splicing variants have been reported in *EIF2B* genes so far,^15^, ^16^, ^17^, ^18^ of which only one is noncanonical, although it was not functionally validated.^15^ Thus, our work is the first functional validation report of a novel, noncanonical splicing mutation in *EIF2B5* associated with ovarioleukodystrophy. In this case, the c.1156 + 13G>A hypomorphic mutation generates a novel splice site 10 bp beyond, that competes with the wild‐type splicing donor site, therefore generating two coexisting populations of transcripts, the mutated, and the WT form. Our work has benefited from the capacity of the minigene assay to quantify the mRNA production in an allele‐specific manner. Indeed, minigene splicing assays have several advantages, such as: (1) circumventing use of patient’s RNA if sample is not available; (2) analysis and quantification of the splicing outcome of mutant alleles without interference of the wild‐type allele; (3) high reproducibility of results; and (4) testing of variants located in any human disease gene, independently of gene expression. This approach allowed us to observe that the cryptic splice site activated by c.1156 + 13G>A is moderately functional in the wild‐type allele, and also that the mutated allele expresses a residual amount of wild‐type mRNA. Competition between the wild‐type and the cryptic splice donor site could determine the expression of some degree of functional EIF2B5 protein and consequently, the mild phenotype exhibited by the patient. Our finding of a partial mis‐splicing is important in view of potential therapies since modulation of splice processes represents a therapeutic approach for some genetic diseases.^19^

Even if ovarioleukodystrophy seems to be an infrequent cause of pure premature ovarian failure (POF),^20^ identification of hyperprolactinemia or amenorrhea should prompt both an exhaustive neurological examination and, possibly, the performance of a cranial MRI. If clinical and neuroimaging studies are suggestive, WES or even WGS are recommended to detect intronic mutations in *EIF2B* genes that could be responsible for mild phenotypes.

## Conflict of Interest

The authors declare that this article was conducted in the absence of any commercial or financial relationships that could be construed as a potential conflict of interest.

## Supporting information


**Data S1.** In silico splicing predictors for variant c.1156+13G>A.
**Figure S1.** Single‐nucleotide splicing variants annotated in ClinVar.
**Figure S2.** Mini‐gene splicing analysis of *EIF2B5* c.1156 + 13 G> A variant.
