# Supplementary material for: A novel hypomorphic splice variant in EIF2B5 gene is associated with mild ovarioleukodystrophy
Source: Ann Clin Transl Neurol. 2020 Aug 15;7(9):1574–9. doi: 10.1002/acn3.51131 (PMC7480926; doi:10.1002/acn3.51131)

SUPPLEMENTAL DATA

a) Supplemental methods

Genetic analyses

Total DNA was extracted from 3 ml of blood using Puregene kit (Qiagen) according to the manufacturer's instructions.

Sanger sequencing

The variant identified in the exome sequencing was validated by PCR direct sequencing. The primer pairs for gDNA sequencing were: 5'-CATTTCCTCTGGTGTGTGGA-3' and 5'-GTGTGCCTG TAATCCCTGCT-3' for the mutation at exon 5 and 5'-GGGGCATTCTTCCTGTCTTT-3' and 5'-TCCCTGTTTCCTCCTTTCCT-3' at intron 7. The primer pair for sequencing *EIF2B5* cDNA was: 5'-ATGTGCTCCTGGGCTCTG-3' and 5'-CGTTCCTTGACCTCAGCATT-3'. Sequence reads were aligned to the genome reference sequences using the program Sequencher.

Plasmid constructs and site-directed mutagenesis

The pSplice*POLR2G* vector (pSP) was digested with EcoRI restriction enzyme (New England Biolabs, Evry, France) and column-purified (Amicon Ultra-0.5 Centrifugal Filter Unit with Ultracel-100 membrane, Millipore, Molsheim, France). *EIF2B5* inserts were PCR-amplified with *Taq* Polymerase (HotStarTaq Master Mix, Qiagen, Courtaboeuf, France), 0.5 µM of each primer (5'-AGGAACATGCGAATTCGGGGACAGGAGGAACAGTAC-3' and 5'- GGAGGCTCAGGAATTC ATCCAATGAGAGGCCCACAG-3'), and ~50 ng of the respective genomic DNAs. PCR conditions were as follow: enzyme activation at 95°C for 15 min, followed by 40 cycles of denaturation at 95°C for 30 sec, annealing at 60°C for 30 sec, extension at 72°C for 30 sec; and a final extension at 72°C for 10 min. Subcloning was carried out by homologous recombination (In-Fusion HD Cloning Kit, Clontech, purchased from Ozyme, Saint-Quentin-en-Yvelines, France) by following the manufacturer instructions. Two microliters of Cloning Enhancer (Clontech) were mixed with 5 µL of unpurified PCR product (i.e., *EIF2B5* insert), incubated at 37°C for 20 min, and then at 80°C for 15 min. One microliter of this product was mixed with ~100 ng of EcoRI-linearized pSP vector and 1X In-Fusion HD Enzyme Premix (Clontech). The reaction was incubated at 50°C for 15 min and placed on ice prior to transformation into competent bacteria (Stellar Competent Cells, Clontech) as described by the manufacturer.

Minigene splicing assay

One microgram of all minigene vectors was transfected with 2 µL of a commercial transfection reagent (Lipofectamine 2000, Life Technologies, Saint-Aubin, France) into the human embryonic kidney (HEK) 293T/17 cells (ATCC Number CRL-11268), COS-7 (ATCC Number CRL-1651) and astrocytoma U-251 MG (Sigma-Aldrich 09063001-1VL) in six-well plates, following the manufacturer protocol. Forty-eight hours after transfection, total RNA was extracted and purified (RNeasy Mini Kit, Qiagen), eluted with 30 µL of RNase-free water and ~100-200 ng of total RNA served as a template for a RT-PCR (OneStep RT-PCR Kit, Qiagen) in a 20-µL final volume with 0.4 µM of both forward and reverse primers in the following conditions: reverse-transcription at 50°C for 30 min, enzyme activation at 95°C for 15 min, followed by 40 cycles of denaturation at 95°C for 30 sec, annealing at 58°C for 30 sec, extension at 72°C for 1 min; and a final extension at 72°C for 10 min. RT-PCR products were loaded on an agarose gel for sizing and gel-purified for direct sequencing when possible. Alternatively gel-purified PCR products were sequenced by Sanger.

Fluorescent PCR

Semi-quantitative fluorescent RT-PCRs were done in triplicate with one FAM-labeled vector primer. 1/20 of cDNA product was mixed in a 20-µL final volume with 0.4 µM of both forward, reverse, and universal FAM primers in the following conditions: enzyme activation at 95°C for 15 min, followed by 32 cycles of denaturation at 94°C for 30 sec, annealing at 58°C for 30 sec, extension at 72°C for 1 min; and a final extension at 72°C for 10 min. Then, 1 µl of PCR product was mixed with 15 μl of Hi-Di Formamide (Life Technologies) and 0.5 μl of Genescan 500 Rox. Samples were run on an ABI3130 sequencer and analyzed with Peak Scanner (Life Technologies). Mean peak areas of each transcript and standard deviations were calculated.

RT-PCR

Total RNA from PBMC was extracted using RNeasy Kit (Qiagen) according to the manufacturer's instructions. One microgram of RNA was retrotranscribed into cDNA using Superscript II reverse transcription reagents in a final volume of 25 µl (Invitrogen). Reactions were performed using 2 µl of 1/20 diluted cDNA in a final volume of 50 µl containing PCR buffer (Sigma 10X with 15mM of $MgCl_2$), 10 pmol of each primer, 250 nmol of each dNTP and 1 unit of Taq polymerase (Sigma). After a denaturation step at 95ºC for 5 min, 38 cycles were programmed as following: 30 s at 95ºC, 30 s at the annealing temperature (56ºC) and 30 s at 72ºC. Forward (5'-AAAATGTGCTCCTGGGCTCT-3') and reverse (5'-CGTTCCTTGACCTCAGCATT-3') primers

were located at exon 7 and 8, respectively. Amplification products were visualized after 2 h of electrophoresis at 60 V on a 4% agarose gel.

b) Supplemental results

Supplemental Table 1

Splicing Predictor	Human Splicing Finder	Max Ent	NetGene2	NN Splice	SpliceView	FSplice
**Result**	Probably no impact on splicing	Novel splice site 6.92>9.46	Novel donor splice site	Donor splice site 0.75>0.95	No changes	Donor splice site 8.48>11

Supplemental Figure 1.

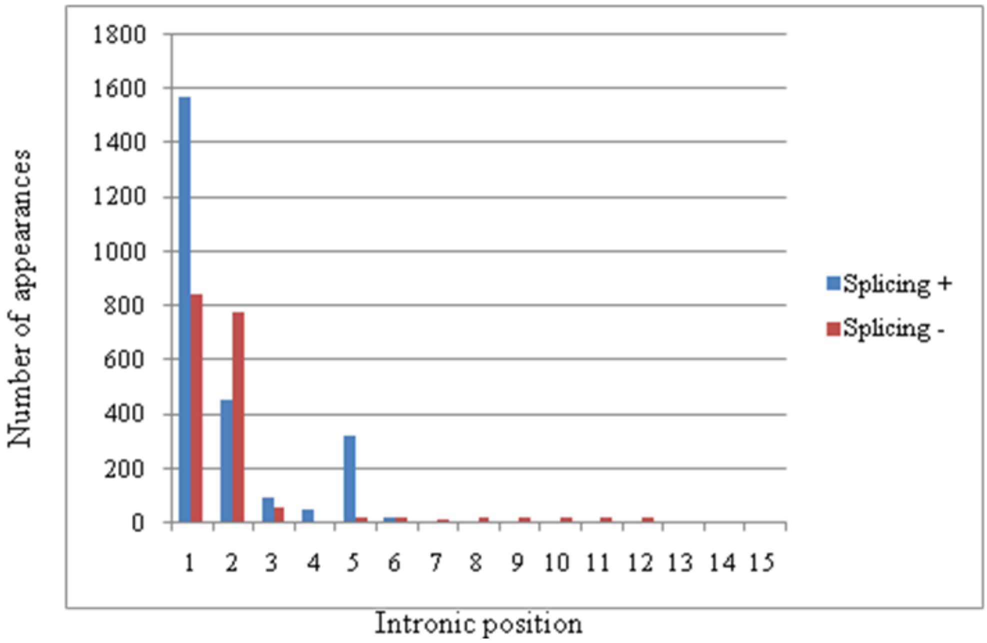

Supplemental Figure 2.

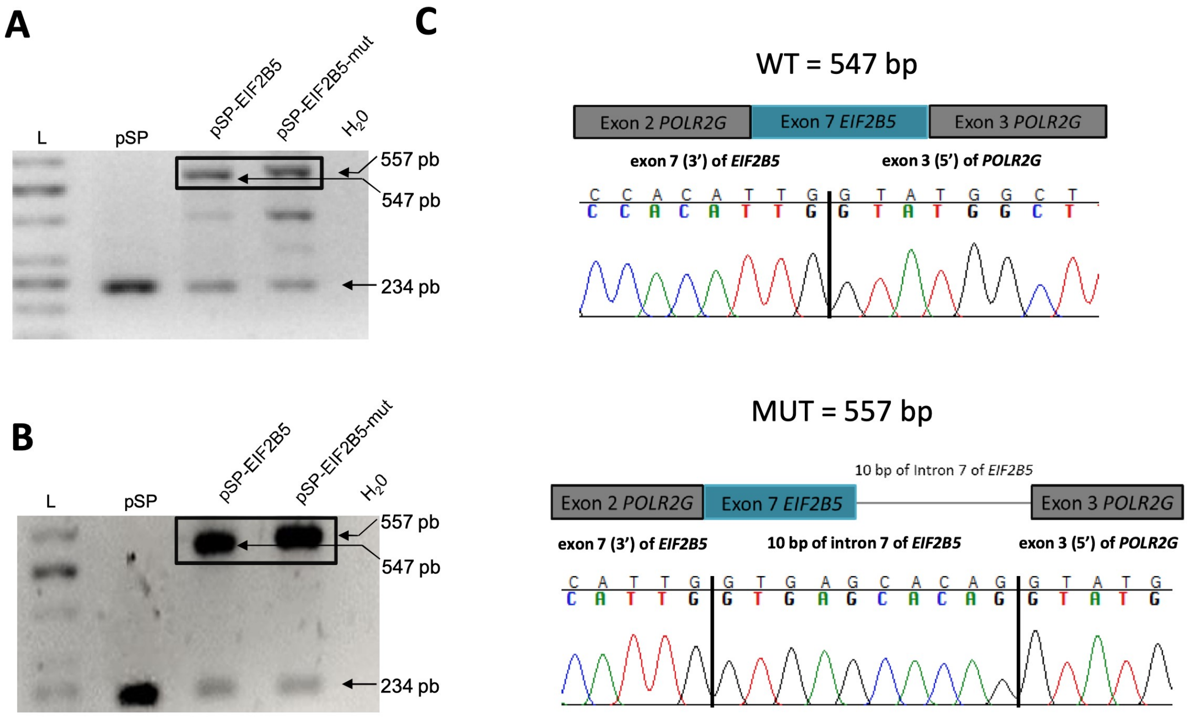

Supplement: Supplementary file 1 — Data S1. In silico splicing predictors for variant c.1156+13G>A. Figure S1. Single‐nucleotide splicing variants annotated in ClinVar. Figure S2. Mini‐gene splicing analysis of EIF2B5 c.1156 + 13 G> A variant. [file ACN3-7-1574-s001.pdf]
